# Cross-sectional study for the clinical application of extracorporeal membrane oxygenation in Mainland China, 2018

**DOI:** 10.1186/s13054-020-03270-1

**Published:** 2020-09-11

**Authors:** Wei Cheng, Xu-Dong Ma, Long-Xiang Su, Huai-Wu He, Lu Wang, Bo Tang, Wei Du, Yuan-kai Zhou, Hao Wang, Na Cui, Yun Long, Da-Wei Liu, Yan-Hong Guo, Ye Wang, Guang-Liang Shan, Xiang Zhou, Shu-Yang Zhang, Yu-Pei Zhao

**Affiliations:** 1 Department of Critical Care Medicine, Peking Union Medical College Hospital, Chinese Academy of Medical Sciences, 1 Shuaifuyuan, Dongcheng District, Beijing, China; 2 Department of Medical Administration, National Health Commission of the People’s Republic of China, Beijing, 100044 China; 3 Department of Epidemiology and Biostatistics, Institute of Basic Medicine Sciences, Chinese Academy of Medical Sciences (CAMS) & School of Basic Medicine, Peking Union Medical College, Beijing, 100730 China; 4 Department of Cardiology, Peking Union Medical College Hospital, Chinese Academy of Medical Sciences, 1 Shuaifuyuan, Dongcheng District, Beijing, China; 5 Department of General Surgery, Peking Union Medical College Hospital, Chinese Academy of Medical Sciences, 1 Shuaifuyuan, Dongcheng District, Beijing, China

**Keywords:** Extracorporeal membrane oxygenation, Epidemiology, Mortality, Complication, China

## Abstract

**Background:**

To investigate the epidemiology and in-hospital mortality of veno-venous (VV) and veno-arterial (VA) extracorporeal membrane oxygenation (ECMO) in Mainland China throughout 2018.

**Methods:**

Patients supported by ECMO from 1700 tertiary hospitals in 31 provinces from January 1 to December 31, 2018, were selected from the National Clinical Improvement System database.

**Results:**

The 1700 included hospitals had 2073 cases of ECMO in 2018, including 714 VV and 1359 VA ECMOs. The average patient age was 50 years (IQR 31–63), and 1346 were male. The average hospital stay was 17 days (IQR 7–30), and the average costs per case was $36,334 (IQR 22,547–56,714). The three provinces with the highest number of ECMO cases were Guangdong, Beijing, and Zhejiang; the southeast coastal areas and regions with higher GDP levels had more cases. Overall in-hospital mortality was 29.6%. Mortality was higher among patients who were male, over 70 years old, living in underdeveloped areas, and who were treated during the summer. Mortality in provinces with more ECMO cases was relatively low. The co-existence of congenital malformations, blood system abnormalities, or nervous system abnormalities increased in-hospital mortality.

**Conclusions:**

Mortality and medical expenses of ECMO among patients in China were relatively low, but large regional and seasonal differences were present. Risk factors for higher in-hospital mortality were older age, male sex, in underdeveloped areas, and treatment during the summer. Additionally, congenital malformations and blood system and nervous system abnormalities were associated with in-hospital mortality.

## Background

Extracorporeal membrane oxygenation (ECMO) was first used in neonates in the 1970s. ECMO has been widely used to treat various types of acute cardiogenic shock and respiratory failure among adults in whom conventional life support has failed. In recent years, an increasing number of patients with severe conditions have benefited from ECMO [1, 2]. Extracorporeal life support technology has developed rapidly worldwide. From 2002 to 2012, the Extracorporeal Life Support Organization (ELSO, Ann, Arbor, MI, USA) registered 2699 cases of veno-arterial (VA) ECMO [3]; in the subsequent 5 years, this number increased to more than 5000 cases [4]. In Asia, there were 5263 cases of VA ECMO in Japan alone between 2010 and 2013 [5]. Because China has the largest population worldwide, the improvement of ECMO in China has clearly increased. In Mainland China [6], the number of ECMO cases and centers has increased yearly, but as of yet there has been no accurate and objective statistical data detailing the epidemiology and patient prognosis of ECMO.

The database of the National Clinical Improvement System (NCIS) of the National Health Commission of the People’s Republic of China (https://ncisdc.medidata.cn/login.jsp) was designed to collect detailed ICU-level data. In 2018, the front page information of patient medical records from 1700 tertiary hospitals was first included in this database. We screened all the available information of ECMO-supported patients who were included in the database and conducted a summary analysis. This work provides a detailed nationwide epidemiological study of ECMO and its associated mortality based on real-world data in Mainland China and provides information to support and improve the use of ECMO.

## Methods

### Patients and study design

In the NCIS system, we searched the front page medical records of all patients from the 1700 tertiary hospitals. The inclusion criteria for patient enrollment were any of the following: (1) diagnosed with ECMO in the discharge diagnosis; (2) operation code of ECMO, 39.6500; (3) catheterization for ECMO noted in the surgical section; and (4) support, monitoring, or replacement of ECMO noted in the surgical section. At the same time, we searched the database for the incidence and type of complications, and whether the prone position was used during ECMO support.

### Study methods

For all the ECMO patients, we collected all information including sex, age, place of residence, diagnosis at discharge, date of hospitalization, discharge date, hospitalization costs, and in-hospital mortality. We then identified the type of ECMO support received by the included patients. If not clearly recorded, the type of ECMO support was determined according to the main diagnosis and operation site. For example, catheterization of the femoral and internal jugular veins was categorized as veno-venous (VV) ECMO; catheterization of the femoral vein and femoral artery or internal jugular vein and femoral artery was categorized as VA ECMO. VV ECMO was determined for patients with a primary diagnosis of acute respiratory failure, and VA ECMO for patients with cardiogenic shock or cardiac arrest.

We classified patients by age as follows: less than 14, 14–20, 21–30, 31–40, 41–50, 51–60, 61–70, and older than 70 years. The 31 provinces/municipalities/autonomous regions of Mainland China were included in this survey (data from Hong Kong, Taiwan, and Macao were not included).

China is divided into seven geographic regions: Central China, North China, East China, South China, Northwest China, Northeast China, and Southwest China. According to its geographical regions and economic development levels, China is also divided into three economic zones: southeastern coastal areas, central inland areas, and western remote areas. The level of gross domestic product (GDP) in China can be divided into high-, middle- and low-GDP regions (eTable 1).

Four seasons were distinguished: spring is from March to May, summer from June to August, autumn from September to November, and winter from December to February.

The costs are expressed in USD, and the exchange rate between RMB and USD was based on the standard of January 1, 2018 (1 USD to 6.5063 RMB).

### Statistical analysis

Normally distributed data are expressed as the mean and standard deviation and were compared using Student’s *t* test. Non-normally distributed data are presented as the median and interquartile range (IQR) and were analyzed using the non-parametric Mann–Whitney *U* test. Categorical variables are expressed as number and percentage and were compared with the chi-square test or Fisher’s exact test. Univariate and multivariate logistic regression analyses were performed successively to determine independent risk factors for in-hospital mortality, considering all variables with *P* < 0.05 in the univariate analysis as significant. The results are expressed as the *P* value and odds ratio (OR) with the 95% confidence interval (CI). IBM SPSS 23.0 software was used for all statistical analyses (IBM Corp., Armonk, NY, USA).

## Results

The NCIS database included front page information of 79,668,156 patients admitted to the 1700 tertiary hospitals of Mainland China in 2018, and a total of 2073 ECMO procedures were conducted, including 1359 (34.4%) VA and 714 (65.6%) VV ECMO procedures (eFigure 1). Dividing by the 1.395 billion residents in China in 2018, the incidence of ECMO was calculated to be 0.148/100,000 inhabitants/year.

### Patient characteristics

The median age of all the ECMO-supported patients was 50 years (IQR 31–63), and 1346 were male, accounting for 64.9%. Children under 14 years accounted for 11.4%, and most patients were between ages 41 and 70. There were significant differences in the age composition between the VA and VV ECMO groups. Most patients who received VA ECMO were 51–60 years old (40.7%) and most who received VV were 41–50 or 61–70 years old (40.6% together). The median length of hospital stay was 17 days (IQR 7, 30), with a significant difference between VV and VA ECMO (16 [IQR 7, 29] vs. 17 [8, 32] days, *P* = 0.036). The median hospitalization cost of ECMO was $36,334 (IQR 22,547–56,714), and VV cost less than VA ECMO ($35,166 vs. $39,162 per patient, *P* < 0.0001; Table 1).

**Table 1:** Baseline characteristics of ECMO patients

	VA ECMO *N* = 1359		VV ECMO *N* = 714		*P*	All *N* = 2073	
	*N*	%	*N*	%		*N*	%
Sex					0.1122		
Male	866	63.7	480	67.2		1346	64.9
Female	493	36.3	234	32.8		727	35.1
Age (years) median, IQR	51	31–63	48	32–63	0.1536	50	31–63
Age (years)					< 0.0001		
< 14	171	12.6	65	9.1		236	11.4
14–20	54	3.9	28	3.9		82	3.9
21–30	112	8.2	72	10.1		184	8.9
31–40	131	9.6	104	14.6		235	11.3
41–50	194	14.3	136	19.1		330	15.9
51–60	277	20.4	98	13.7		375	18.1
61–70	276	20.3	154	21.6		430	20.7
> 70	144	10.6	57	7.9		201	9.7
Hospital stay (days) median, IQR	16	7–29	17	8–32	0.0360	17	7–30
Hospital stay (days)					0.0995		
1–10	455	33.5	232	32.5		687	33.1
11–20	365	26.9	171	23.9		536	25.9
21–30	229	16.9	114	15.9		343	16.6
> 30	310	22.8	197	27.6		507	24.5
Overall cost (USD) median, IQR	35,166	22,056-53,148	39,162	24,130-64,491	< 0.0001	36,334	22,547–56,714
Overall cost (USD)					0.0002		
< 15,370	190	13.9	78	10.9		268	12.9
15,370–	389	28.6	164	22.9		553	26.7
30,740–	348	25.6	180	25.2		528	25.5
46,110–	432	31.8	292	40.9		724	34.9

*ECMO* extracorporeal membrane oxygenation, *VV* veno-venous, *VA* veno-arterial, *IQR* interquartile range, *USD* US dollar

*P* value for the comparison between VV and VA ECMO

### Geographic characteristics

**Fig. 1:**
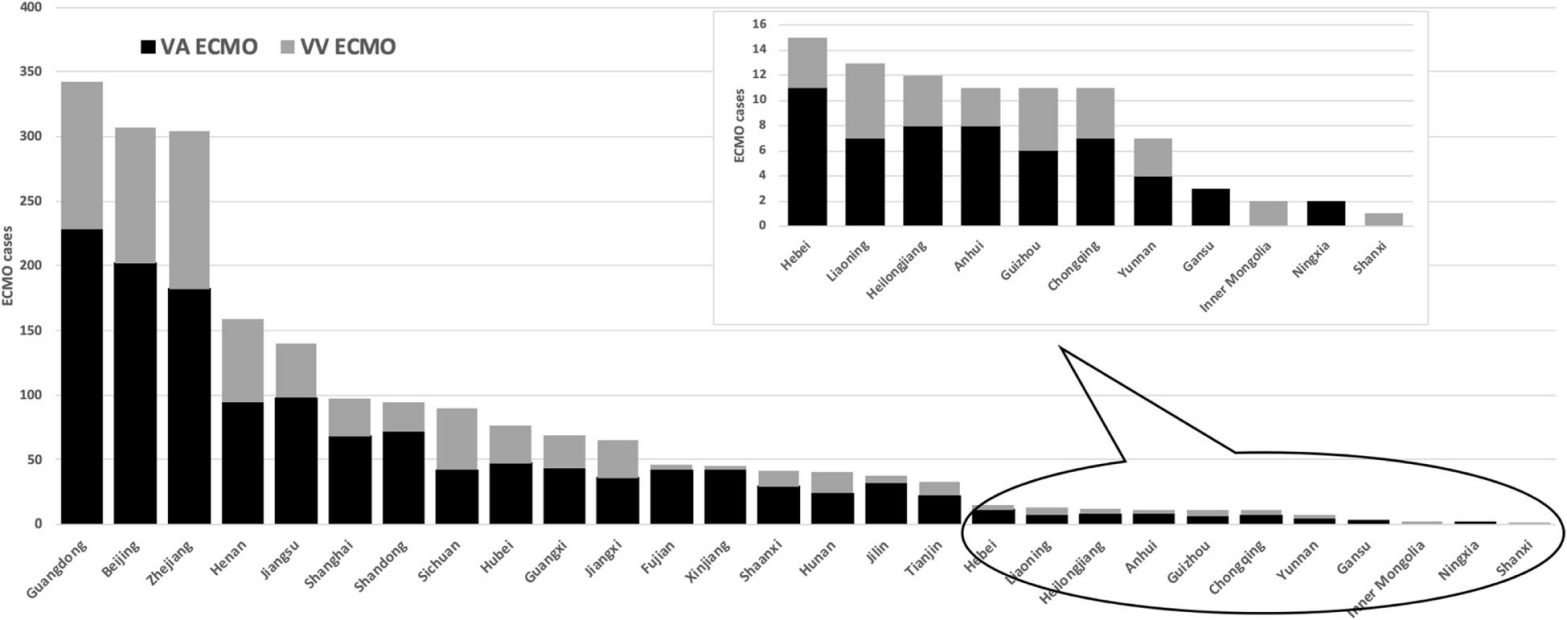
Number of VV and VA ECMO patients in all the provinces of China. ECMO, extracorporeal membrane oxygenation; VV, veno-venous; VA, veno-arterial

Except for Hainan, Qinghai, and Tibet, 28 of the 31 provinces have implemented ECMO support for hospitalized patients. More than 300 cases of ECMO were conducted in Guangdong, Beijing, and Zhejiang, whereas there were fewer than five ECMO procedures conducted in Gansu, Inner Mongolia, Ningxia, and Shanxi. The regional differences between VV and VA ECMO were roughly the same (Fig. 1 and eTable 2).

In terms of geographical area, the region with the largest number of ECMO cases was East China, accounting for 36.5%. The four regions of North, East, South, and Central China together accounted for 90%; the remaining three regions only accounted for 10% of the total cases (Table 2).

**Table 2:** The number of ECMO cases and in-hospital mortality in different gender, age group, geographic, and economic regions

	All patients		VA ECMO		VV ECMO		***P***
	*N*	Mortality N (%)	*N*	Mortality N (%)	*N*	Mortality N (%)	
**Genders**							
Male	1346	420 (31.2)	866	278 (32.1)	480	142 (29.6)	0.339
Female	727	194 (26.7)	493	128 (25.9)	234	66 (28.2)	0.523
**Age groups**							
< 14	236	72 (30.5)	171	52 (30.4)	65	20 (30.8)	0.957
14–20	82	28 (34.2)	54	19 (35.2)	28	9 (32.1)	0.783
21–30	184	48 (26.1)	112	30 (26.8)	72	18 (25.0)	0.788
31–40	235	59 (25.1)	131	33 (25.2)	104	26 (25.0)	0.973
41–50	330	87 (26.4)	194	56 (28.9)	136	31 (22.8)	0.218
51–60	375	115 (30.7)	277	81 (29.2)	98	34 (34.7)	0.314
61–70	430	131 (30.5)	276	86 (31.2)	154	45 (29.2)	0.675
> 71	201	74 (36.8)	144	49 (34.0)	57	25 (43.9)	0.193
**Geographical area of China**							
East China	757	195 (25.8)	506	139 (27.5)	251	56 (22.3)	0.126
South China	411	156 (37.9)	271	98 (36.2)	140	58 (41.4)	0.297
North China	358	120 (33.5)	235	77 (32.8)	123	43 (34.9)	0.676
Central China	275	59 (21.5)	165	37 (22.4)	110	22 (20)	0.631
Southwest	119	44 (36.9)	59	26 (44.1)	60	18 (27.3)	0.112
Northwest	91	20 (21.9)	76	16 (21.1)	15	4 (26.7)	0.631
Northeast	62	20 (32.3)	47	13 (27.7)	15	7 (46.7)	0.170
**Economic zones of China**							
Southeast coastal area	1471	466 (31.7)	982	310 (31.6)	489	156 (31.9)	0.897
Central inland area	403	88 (21.8)	249	57 (22.9)	154	31 (20.1)	0.514
Western remote area	199	60 (30.2)	128	39 (30.5)	71	21 (29.6)	0.896
**Different GDP level areas of China**							
High	1441	441 (30.6)	961	289 (30.1)	480	152 (31.7)	0.536
Middle	438	105 (23.9)	279	68 (24.4)	159	37 (23.3)	0.795
Low	194	68 (35.1)	119	49 (41.2)	75	19 (25.3)	0.024
**Total**	2073	614 (29.6)	1359	406 (29.9)	714	208 (29.1)	0.725

*ECMO* extracorporeal membrane oxygenation, *VV* veno-venous, *VA* veno-arterial, *Non-surv* non-survivors, *mort* in-hospital mortality, *Mortality N* (*%*)the number and proportion of the non-survivors; *GDP* gross domestic product

*P* value for the comparison of non-survivors of the VA and VV ECMO patients

The number of ECMO cases was significantly related to the local economic situation, which gradually decreased from the developed southeast coastal economic belt to the relatively undeveloped remote western areas and from areas with high GDP to those with low GDP (Table 2).

Through our analysis, we found a significant seasonal difference in the number of ECMO procedures. VA ECMO was mainly conducted in autumn and winter (55.1%), and VV ECMO was mainly conducted in winter and spring (64.2%; eFigure 2 and eTable 3).

### Outcomes and risk factors

The in-hospital mortality among all ECMO patients was 29.6%, with 29.9% for VA and 29.1% for VV. Additionally, the mortality rate of male patients was higher than that of females (31.2% vs. 26.7%). The mortality differed slightly according to age group, with a higher rate among patients between age 14–20 years and those over 70 years; the lowest mortality rate was among those aged 30–40 years (Table 2).

**Fig. 2:**
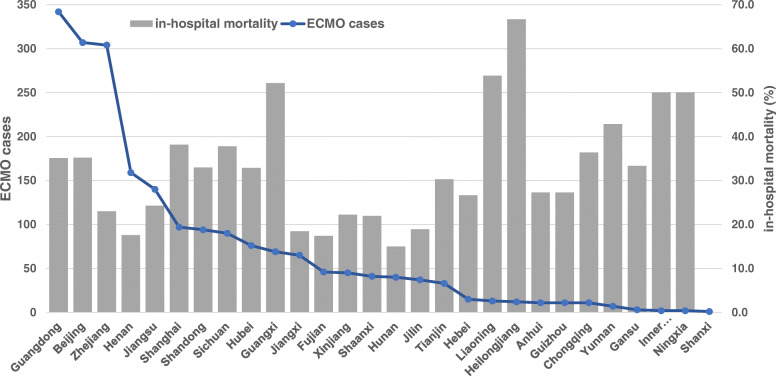
In-hospital mortality of ECMO patients in different provinces

We found large differences in in-hospital mortality for ECMO patients in different provinces, with the highest mortality reaching 53% and the lowest being 15% (Fig. 2 and eTable 2). The highest in-hospital mortality rates among patients receiving ECMO were in South and Southwest China (38.0% and 37.0%, respectively), and the lowest were in Northwest and Central China (22.0% and 21.5%, respectively). The in-hospital mortality of patients receiving ECMO in different economic zones and regions with different GDP levels also differed (Table 2).

The in-hospital mortality of ECMO patients across the seasons also varied. In spring and summer, the number of ECMO cases was lower and the mortality relatively higher, while in autumn and winter, the number of ECMO cases was higher and the mortality relatively lower (eFigure 2 and eTable 3). Mortality rate for both VV and VA ECMO showed this temporal distribution.

Using the baseline data and comorbidities of ECMO patients, we performed Cox multivariate analysis to elucidate the risk factors of in-hospital mortality. ECMO performed during the summer, patients aged over 70 years, and male sex were independent risk factors for higher in-hospital mortality. Congenital malformations, blood system diseases, and neurological diseases were also independent risk factors. Residing in areas with middle-GDP levels was a protective factor (Table 3).

**Table 3:** Risk factor analysis for in-hospital mortality of ECMO patients

Factors	All			
	OR	95% CI		*P*
Type (Ref = VA)				
VV	0.95	0.75	1.19	0.6431
Age (Ref = 0–13)				
14–20	1.11	0.62	1.98	0.7255
21–30	0.84	0.52	1.35	0.4699
31–40	0.80	0.50	1.27	0.3469
41–50	0.87	0.56	1.34	0.5280
51–60	1.13	0.74	1.71	0.5823
61–70	1.12	0.74	1.70	0.5782
> 71	1.61	1.01	2.55	0.0452
Sex (Ref = male)				
Female	0.80	0.65	0.99	0.0400
GDP (Ref = high)				
Middle	0.72	0.56	0.93	0.0110
Low	1.19	0.86	1.66	0.3017
Season (Ref = spring)				
Summer	1.35	1.01	1.81	0.0420
Autumn	0.92	0.69	1.22	0.5658
Winter	1.10	0.84	1.46	0.4858
Diagnosis at discharge				
Infectious disease	1.01	0.79	1.29	0.9584
Blood system disease	1.29	1.01	1.65	0.0413
Endocrine disease	1.18	0.96	1.46	0.1221
CNS disease	1.85	1.40	2.43	< 0.0001
Circulatory disease	–	–	–	–
Respiratory disease	–	–	–	–
Digestive disease	1.10	0.88	1.38	0.3953
Urogenital diseases	1.04	0.83	1.31	0.7348
Congenital malformations	1.59	1.08	2.33	0.0180
Injury and poisoning	1.12	0.83	1.52	0.4607

*ECMO* extracorporeal membrane oxygenation, *VV* veno-venous, *VA* veno-arterial, *OR* odd ration, *95%CI* 95% confidence interval, *GDP* gross domestic product, *CNS* disease central nervous disease

### Complications

The most common complications during ECMO treatment were hemorrhage, infection, and mechanical complications. The proportion of bleeding complications was as high as 23.2%. Infection complications occurred in 15.1% of patients receiving ECMO, with bloodstream infection accounting for 9.6%. The incidence of ECMO-related mechanical complications was 2.2%, including blockage, accidental decannulation, and hemolysis. There were some differences in the types and incidence of complications among the different regions and among the ECMO centers (eTable 4 and eTable 5).

We also determined the number of patients treated with VV ECMO combined with the prone position. In provinces with more than 25 cases of VV ECMO, the average proportion of patients treated in the prone position was 39.5%, and the highest was 83.3% (eTable 6).

## Discussion

This is the first detailed nationwide epidemiologic study of ECMO and its associated mortality based on medical front page data in Mainland China. The data obtained in this study are authentic and can well reflect the current use and in-hospital mortality of ECMO.

Previous statistics in China [6] have shown that the number of ECMO cases has been increasing each year, as have the number of ECMO centers, which indicates that ECMO is developing rapidly. Our dataset indicated that the incidence of ECMO among the Chinese population was 0.148/100,000 inhabitants/year. These data from 1700 tertiary hospitals might not include all 2018 instances of ECMO in the Mainland, as of all the 2451 tertiary hospitals that were in operation in 2018, the rest hospitals might have also performed this technique in addition to these ones, although to a much lesser extent. In a non-SARS-CoV-2 world, a reasonable figure for ECMO incidence would be 0.3–0.5/100,000 if the resources are used wisely [1, 7–11] . China still has a large gap compared with this figure. However, regarding their geographical distribution, ECMO centers have been established in many locations throughout China, showing a trend of rapid development. Owing to different levels of economic development, the improvement and proficiency of ECMO centers throughout China also varies. For example, there were 342 ECMO patients in the most developed area, whereas there was only one patient in the least developed area, which is a dramatic difference. In relation to this, there were also significant differences found for in-hospital mortality. Given the imbalance of medical resources, Chinese medical managers are actively organizing the introduction and implementation of ECMO-related norms and conducting ECMO-related medical and nursing training. These measures aim to markedly improve the level of ECMO development and the patient survival rate in Mainland China.

Our data showed that the average length of hospital days on ECMO was 17 days, which is similar to that of other countries [1]. We also found that the hospital stay was significantly different between VA and VV ECMO. However, the average cost was $36,334 (IQR 22,547–56,714), which is significantly lower than that in other countries. A recent study showed that the average cost for patients receiving ECMO in the ICU in the US was $73,122 for the placement procedures, with a total hospital cost of $210,100 [12]. Unlike in many countries [1], medical insurance in China does not fully cover the costs of ECMO treatment, and patients themselves must pay a large part of the costs. Although China’s GDP per capita reached $9936 in 2018, it was only $1594 in remote areas with low socioeconomic levels. Eliminating the gap between the rich and poor is an important means to promote the balanced development of ECMO in China.

The mortality varied among patients who received ECMO. ELSO data from 2002 to 2012 [3] showed that the total survival-to-discharge rate of VA ECMO was 41%. In 2014, the in-hospital mortalities of German patients treated with VV or VA ECMO were 58% and 66%, respectively [7]. Recently, the reported overall mortality has decreased to approximately 40% [12, 13] (35% in children) [14, 15]. The ECMO mortality rate is also related to the type of disease, and the reported mortality for those with acute myocarditis may be as low as 25% [16]. However, the mortality rates found in large-scale epidemiological studies have all been around 60%. Our data showed that the in-hospital mortality was approximately 30%, which is significantly lower than that in other countries. There might be several reasons for this, listed below.

1. Lack of data on diagnoses and the severity of illness in the medical front pages database. In terms of indications for ECMO, acute myocarditis, acute coronary disease, acute respiratory distress syndrome, and other reversible diseases are common, which would greatly reduce mortality. However, using the present dataset, we could not distinguish the specific primary disease of patients; therefore, additional data are needed for further analysis. At the same time, waiting for organ transplantation with ECMO support is not currently an indication in China.
2. Fewer ECMO complications. Bleeding, infection, and mechanical complications were much lower in our data than those reported by some countries, especially bloodstream infections [8, 17–19]. This could lower the mortality, as more than 10% of the causes of death could be attributed to complications. On the one hand, this might be related to the physical condition of Chinese people who generally have a smaller body size. Furthermore, the proportion of obese people is relatively low, which makes the procedure much smoother and results in fewer complications during ECMO maintenance. Of course, this requires analysis of more specific indicators, such as the body mass index. On the other hand, hospitals conducting ECMO in China are all ECMO centers in each province, and in most centers the procedures are performed and maintained by specialized medical teams. It has been more than 10 years since the first ECMO was performed in China. As treatment has become more sophisticated, centers have gained much more experience. Our results were consistent with those of several large randomized controlled studies conducted in ECMO centers, all showing lower mortality rates [2, 16, 20].
3. More stringent indications. ECMO is not fully covered by national insurance at present; therefore, ECMO candidates are thoroughly evaluated by clinicians, which potentially led to selection bias. Furthermore, family members may be reluctant to authorize this procedure. We found that the median age of ECMO patients was 50 years, and fewer than one third were over 60. In Germany, 50% of ECMO patients in 2014 were over 60 [7]. Numerous studies have reported that age is significantly correlated with mortality [1, 5, 7]; a lower age undoubtedly improves the survival rate of the patients.
4. A high level of care. The establishment and maintenance of ECMO reflects the overall medical level of a hospital, which is highly valued by clinicians and hospital managers. For VV ECMO patients, the proportion of patients treated in the prone position might be as high as 30%, and this might be greater than 80% in certain centers. This more aggressive and advanced treatment could also reduce the mortality rate.
5. Dying at home. The traditional preference of many Chinese people is to die at home. Some patients without hope of further treatment would choose not to die in the hospital. This information is not reflected in the front page of medical records, but it might be reflected in the ECMO mortality rate.

As to additional study findings, the risk of in-hospital mortality was significantly increased in patients older than 70 years, which is consistent with the conclusions of several studies [1, 5, 7]. Seasonal variation in mortality was also identified in this study. The number of ECMO procedures was found significantly different in different seasons, and the proficiency of medical staff might cause this difference in mortality. There might be multiple other complex reasons for these variations [21], and additional data are needed to further analyze of this finding. We also found that blood system and nervous system abnormalities increased the risk of in-hospital mortality, which might be related to hemorrhage, thrombosis, or central nervous system complications [18, 22]. Although we were unable to clearly illustrate such relationships using the existing data, the incidence of bleeding and thrombosis complications were similar to those found in previous studies [23].

### Limitations

This is the first study of ECMO based on objective data in Mainland China. Although the data are objective and detailed, some limitations remain that are directly related to the structural features of the database used. First, the disease categories were not sufficiently detailed to distinguish the survival rate for specific diseases after ECMO support. In the future, further division according to different disease types will help to clarify the effect of ECMO treatment and its most appropriate uses. Second, Smith and El Sibai both found that the treatment duration of ECMO was significantly correlated with in-hospital mortality [1, 3, 7]. We failed to reach a similar conclusion owing to limitations in the data acquisition. In future studies, we hope to include this information. Finally, because this was a retrospective study, it could not be determined whether some cases had complications owing to a lack of sufficient detail in the database. The actual consequences of complications must be further distinguished and perhaps analyzed using a prospective study design.

## Conclusions

ECMO technology is developing rapidly in Mainland China, but a large gap remains in comparison with other countries. From the data analysis, in-hospital mortality was relatively high in older male patients, patients from less-developed areas, and in those treated during the summer. The presence of congenital malformations and abnormalities of the blood and nervous systems also showed increased in-hospital mortality. The large regional and seasonal differences could be because ECMO development in China is uneven. China is in the early stages of ECMO use. Therefore, standardized ECMO training and treatment procedures should be established in China to further improve the use of ECMO.

## Supplementary information

**Additional file 1: eTable 1** regional division of China.doc.

**Additional file 2: eTable 2** the number of ECMO cases and in-hospital mortality in all provinces in mainland China.doc.

**Additional file 3: eTable 3** the number of ECMO cases and in-hospital mortality in different months.

**Additional file 4: eTable 4** ECMO related complications in 7 geographical regions of China.

**Additional file 5: eTable 5** ECMO related complications in provinces with more than 50 cases.

**Additional file 6: eTable 6** The number and proportion of patients treated with prone position in the provinces with more than 25 cases of VV ECMO.

**Additional file 7: eFigure 1** Supplement 2 Flow chart of enrollment. ECMO extracorporeal membrane oxygenation; VV veno-venous; VA veno-arterial.

**Additional file 8: eFigure 2** the number of ECMO cases and in-hospital mortality in different months ECMO extracorporeal membrane oxygenation; VV veno-venous; VA veno-arterial.

## Data Availability

Please contact the authors for data requests.

## Abbreviations

VV and VA ECMO: Veno-venous and veno-arterial extracorporeal membrane oxygenation, respectively
IQR: Interquartile range
OR: Odds ratio
95% CI: Confidence interval
GDP: Gross domestic product
ELSO: The Extracorporeal Life Support Organization
ICU: Intensive care unit
RMB: Chinese money renminbi
USD: US dollar
