# Supplementary material for: Cross-sectional study for the clinical application of extracorporeal membrane oxygenation in Mainland China, 2018
Source: Crit Care. 2020 Sep 11;24:554. doi: 10.1186/s13054-020-03270-1 (PMC7484920; doi:10.1186/s13054-020-03270-1)
Supplement: Supplementary file 1 — Additional file 1: eTable 1 regional division of China.doc. [file 13054_2020_3270_MOESM1_ESM.docx]

**eTable 1 Regional division of China**

| **Geographical area of China** | |
| --- | --- |
| East China | Shanghai City, Jiangsu, Zhejiang, Anhui, Fujian, Jiangxi, and Shandong provinces, as well as Taiwan (not analyzed in this survey) |
| South China | Guangdong Province, Guangxi Zhuang Autonomous Region (Guangxi), Hainan Province, as well as Hong Kong and Macao (not analyzed in the survey) |
| North China | Beijing, Tianjin, Hebei Province, Shanxi Province, and Inner Mongolia Autonomous Region (Inner Mongolia) |
| Central China | Henan, Hunan, and Hubei provinces |
| Southwest | Sichuan, Guizhou, and Yunnan provinces, Chongqing City, and the Tibet Autonomous Region (Tibet) |
| Northwest | Shaanxi, Gansu, and Qinghai provinces, Ningxia Hui Autonomous Region (Ningxia), and the Xinjiang Uygur Autonomous Region (Xinjiang) |
| Northeast | Heilongjiang, Jilin, and Liaoning provinces |
| **Economic zones of China** | |
| Southeast coastal area | Beijing, Tianjin, Hebei, Liaoning, Shanghai, Jiangsu, Zhejiang, Fujian, Shandong, Guangdong, Guangxi, Hainan, and Chongqing |
| Central inland area | Shanxi, Inner Mongolia, Jilin, Heilongjiang, Anhui, Jiangxi, Henan, Hubei, and Hunan |
| Western remote area | Sichuan, Guizhou, Yunnan, Tibet, Shaanxi, Gansu, Qinghai, Ningxia, and Xinjiang |
| **Different GDP level areas of China** | |
| High | Guangdong, Jiangsu, Shandong, Zhejiang, Henan, Sichuan, Hubei, Hunan, Hebei and Fujian Provinces |
| Middle | Shanghai, Beijing, Anhui, Liaoning, Shaanxi, Jiangxi, Chongqing, Guangxi, Tianjin and Yunnan provinces |
| Low | Inner Mongolia, Shanxi, Heilongjiang, Jilin, Guizhou, Xinjiang, Gansu, Hainan, Ningxia, Qinghai and Tibet Provinces |

**All the data are from China's new economic white paper 2018**
