# Supplementary material for: Cross-sectional study for the clinical application of extracorporeal membrane oxygenation in Mainland China, 2018
Source: Crit Care. 2020 Sep 11;24:554. doi: 10.1186/s13054-020-03270-1 (PMC7484920; doi:10.1186/s13054-020-03270-1)
Supplement: Supplementary file 2 — Additional file 2: eTable 2 the number of ECMO cases and in-hospital mortality in all provinces in mainland China.doc. [file 13054_2020_3270_MOESM2_ESM.docx]

eTable 2 **The number of ECMO cases and in-hospital mortality in all provinces in mainland China**

|  | **VA ECMO** | | **VV ECMO** | | **All** | |
| --- | --- | --- | --- | --- | --- | --- |
| **Provinces** | **N** | **mortality（%）** | **N** | **Mortality（%）** | **N** | **Mortality（%）** |
| **Guangdong** | 228 | 31.1 | 114 | 42.9 | 342 | 35.1 |
| **Peking** | 202 | 33.7 | 105 | 35.2 | 307 | 34.2 |
| **Zhejiang** | 182 | 25.8 | 122 | 18.9 | 304 | 23.0 |
| **Henan** | 94 | 17.0 | 65 | 18.5 | 159 | 17.6 |
| **Jiangsu** | 98 | 27.6 | 42 | 16.7 | 140 | 24.3 |
| **Shanghai** | 68 | 39.7 | 29 | 34.5 | 97 | 38.1 |
| **Shandong** | 72 | 27.8 | 22 | 50 | 94 | 32.9 |
| **Sichuan** | 42 | 45.2 | 48 | 31.3 | 90 | 37.8 |
| **Hubei** | 47 | 34.0 | 29 | 31.0 | 76 | 32.9 |
| **Guangxi** | 43 | 62.8 | 26 | 34.6 | 69 | 52.2 |
| **Jiangxi** | 36 | 22.2 | 29 | 13.8 | 65 | 18.5 |
| **Fujian** | 42 | 16.7 | 4 | 25 | 46 | 17.4 |
| **Xinjiang** | 42 | 16.7 | 3 | 66.7 | 45 | 20 |
| **Shaanxi** | 29 | 24.1 | 12 | 16.7 | 41 | 21.9 |
| **Hunan** | 24 | 20.8 | 16 | 6.3 | 40 | 15 |
| **Jilin** | 32 | 18.8 | 5 | 20 | 37 | 18.9 |
| **Tianjin** | 22 | 27.3 | 11 | 36.4 | 33 | 30.3 |
| **Hebei** | 11 | 27.3 | 4 | 25 | 15 | 26.7 |
| **Liaoning** | 7 | 57.1 | 6 | 50 | 13 | 53.9 |
| **Heilongjiang** | 8 | 37.5 | 4 | 75 | 12 | 50 |
| **Anhui** | 8 | 37.5 | 3 | 0 | 11 | 27.3 |
| **Guizhou** | 6 | 50 | 5 | 0 | 11 | 27.3 |
| **Chongqing** | 7 | 42.9 | 4 | 25 | 11 | 36.4 |
| **Yunnan** | 4 | 25 | 3 | 66.7 | 7 | 42.9 |
| **Gansu** | 3 | 33.3 | 0 | - | 3 | 33.3 |
| **Inner Mongolia** | 0 | - | 2 | 50 | 2 | 50 |
| **Ningxia** | 2 | 50 | 0 | - | 2 | 50 |
| **Shanxi** | 0 | - | 1 | 0 | 1 | 0 |

ECMO extracorporeal membrane oxygenation; VV veno-venous; VA veno-arterial.
