# Supplementary material for: Cross-sectional study for the clinical application of extracorporeal membrane oxygenation in Mainland China, 2018
Source: Crit Care. 2020 Sep 11;24:554. doi: 10.1186/s13054-020-03270-1 (PMC7484920; doi:10.1186/s13054-020-03270-1)
Supplement: Supplementary file 3 — Additional file 3: eTable 3 the number of ECMO cases and in-hospital mortality in different months. [file 13054_2020_3270_MOESM3_ESM.docx]

**eTable 3 The number of ECMO cases and in-hospital mortality in different months**

|  | VA ECMO | | VV ECMO | | All | |
| --- | --- | --- | --- | --- | --- | --- |
| Month | N | mortality（%） | N | Mortality（%） | N | Mortality（%） |
| 1 | 107 | 36.5 | 140 | 27.9 | 247 | 31.6 |
| 2 | 77 | 29.9 | 100 | 21.0 | 177 | 24.9 |
| 3 | 112 | 24.1 | 55 | 23.6 | 167 | 23.9 |
| 4 | 90 | 28.9 | 45 | 33.3 | 135 | 30.4 |
| 5 | 110 | 30.0 | 46 | 39.1 | 156 | 32.7 |
| 6 | 97 | 36.1 | 50 | 38.0 | 147 | 36.7 |
| 7 | 96 | 34.4 | 31 | 41.9 | 127 | 36.2 |
| 8 | 106 | 35.9 | 46 | 28.3 | 152 | 33.6 |
| 9 | 134 | 23.1 | 40 | 22.5 | 174 | 22.9 |
| 10 | 154 | 27.9 | 38 | 34.2 | 192 | 29.2 |
| 11 | 144 | 28.5 | 51 | 27.5 | 195 | 28.2 |
| 12 | 132 | 28.0 | 72 | 29.2 | 204 | 28.4 |

ECMO extracorporeal membrane oxygenation; VV veno-venous VA veno-arterial
