# Supplementary material for: Cross-sectional study for the clinical application of extracorporeal membrane oxygenation in Mainland China, 2018
Source: Crit Care. 2020 Sep 11;24:554. doi: 10.1186/s13054-020-03270-1 (PMC7484920; doi:10.1186/s13054-020-03270-1)
Supplement: Supplementary file 4 — Additional file 4: eTable 4 ECMO related complications in 7 geographical regions of China. [file 13054_2020_3270_MOESM4_ESM.docx]

**eTable 4 ECMO related complications in 7 geographical regions of China**

|  | all | East | South | North | Central | Southwest | Northwest | Northeast |
| --- | --- | --- | --- | --- | --- | --- | --- | --- |
| N | 2073 | 757 | 411 | 358 | 275 | 119 | 91 | 62 |
| bleeding（%）  cerebral（%）  gastrointestinal（%）  Puncture site（%）  pulmonary（%） | 23.2  3.5  7.5  11.2  3.1 | 16.9  1.6  6.3  10.7  2.2 | 14.1  1.2  3.7  7.4  2.4 | 28.1  6.2  9.3  8.0  1.9 | 30.6  3.3  9.2  16.5  3.4 | 42.5  5.4  13.2  34.1  19.8 | 37.1  8.1  11.3  22.6  8.1 | 21.4  9.5  4.8  11.9  2.4 |
| infection（%）  bloodstream（%）  puncture site（%） | 15.11  9.6  1.7 | 10.46  4.9  1.9 | 4.39  1.7  0.7 | 25.1  21.1  0.5 | 17.2  4.9  3.4 | 18.6  13.2  4.2 | 12.9  8.1  6.5 | 7.14  4.8  2.4 |
| Mechanical  hemolysis（%）  blockage（%）  accidental decannulation（%） | 3.88  2.2  1.3  0.4 | 2.28  1.3  0.8  0.2 | 2.27  1.4  0.5  0.4 | 4.13  2.2  1.8  0.2 | 6.83  4.0  1.9  0.9 | 7.17  4.2  1.8  1.2 | 9.67  6.5  3.2  0 | 7.14  2.4  2.4  2.4 |

ECMO extracorporeal membrane oxygenation
