# Supplementary material for: Cross-sectional study for the clinical application of extracorporeal membrane oxygenation in Mainland China, 2018
Source: Crit Care. 2020 Sep 11;24:554. doi: 10.1186/s13054-020-03270-1 (PMC7484920; doi:10.1186/s13054-020-03270-1)
Supplement: Supplementary file 5 — Additional file 5: eTable 5 ECMO related complications in provinces with more than 50 cases. [file 13054_2020_3270_MOESM5_ESM.docx]

**eTable 5 ECMO related complications in provinces with more than 50 cases**

|  | all | Guangdong | Peking | Zhejiang | Henan | Jiangsu | Shanghai | Shandong | Sichuan | Hubei | Guangxi | Jiangxi |
| --- | --- | --- | --- | --- | --- | --- | --- | --- | --- | --- | --- | --- |
| N | 2073 | 342 | 307 | 304 | 159 | 140 | 97 | 94 | 90 | 76 | 69 | 65 |
| bleedingcomplications（%）  cerebral（%）  gastrointestinal  Puncture site（%）  pulmonary（%） | 23.2  3.5  7.5  11.2  3.1 | 12.7  1.0  3.5  6.1  2.4 | 31.8  7.2  10.5  8.6  2.4 | 24.5  1.1  10.9  15.3  4.7 | 31.9  3.0  10.2  15.0  3.6 | 17.7  2.3  6.2  8.5  0.4 | 28.4  2.1  7.4  12.6  4.2 | 31.3  3.6  9.6  26.5  4.8 | 52.2  6.1  14.8  39.1  26.1 | 35.2  1.4  5.6  35.2  4.2 | 8.0  1.3  2.7  4.0  0 | 17.6  6.8  5.4  8.1  1.4 |
| infectioncomplications（%）  bloodstream（%）  puncture site（%） | 15.1  9.6  1.7 | 3.8  1.3  0.6 | 29.6  25.1  0.4 | 17.3  5.9  3.9 | 20.0  5.2  3.2 | 6.9  4.2  0.8 | 12.6  5.3  3.2 | 12.1  7.2  0 | 23.5  17.4  4.4 | 8.5  5.6  4.2 | 10.7  4.0  1.3 | 6.8  2.7  4.1 |
| Mechanical complications  hemolysis（%）  blockage（%）  accidental decannulation（%） | 3.9  2.2  1.3  0.4 | 1.6  0.8  0.4  0.5 | 4.8  2.6  0.2  2.0 | 2.8  1.7  0.3  0.8 | 7.6  4.4  1.2  2.0 | 2.3  2.3  0  0 | 5.3  1.1  0.0  4.2 | 0 | 6.9  5.2  0.9  0.9 | 4.2  2.8  0  1.4 | 2.7  1.3  0.0  1.3 | 4.1  2.7  0  1.4 |

ECMO extracorporeal membrane oxygenation
