# Supplementary material for: Cross-sectional study for the clinical application of extracorporeal membrane oxygenation in Mainland China, 2018
Source: Crit Care. 2020 Sep 11;24:554. doi: 10.1186/s13054-020-03270-1 (PMC7484920; doi:10.1186/s13054-020-03270-1)
Supplement: Supplementary file 6 — Additional file 6: eTable 6 The number and proportion of patients treated with prone position in the provinces with more than 25 cases of VV ECMO. [file 13054_2020_3270_MOESM6_ESM.docx]

**eTable 6 The number and proportion of patients treated with prone position in the provinces with more than 25 cases of VV ECMO**

| **Provinces** | **VV-ECMO** | **N. of Prone position** | **%** |
| --- | --- | --- | --- |
| Zhejiang | 122 | 25 | 20.5% |
| Guangdong | 114 | 11 | 9.7% |
| Peking | 105 | 20 | 19.1% |
| Henan | 65 | 29 | 44.6% |
| Sichuan | 48 | 40 | 83.3% |
| Jiangsu | 42 | 15 | 35.7% |
| Shanghai | 29 | 4 | 13.8% |
| Hubei | 29 | 20 | 69.0% |
| Jiangxi | 29 | 11 | 37.9% |
| Guangxi | 26 | 16 | 61.5% |
| Average |  |  | 39.5% |

VV ECMO veno-venous extracorporeal membrane oxygenation
