# Supplementary material for: Cross-sectional study for the clinical application of extracorporeal membrane oxygenation in Mainland China, 2018
Source: Crit Care. 2020 Sep 11;24:554. doi: 10.1186/s13054-020-03270-1 (PMC7484920; doi:10.1186/s13054-020-03270-1)
Supplement: Supplementary file 7 — Additional file 7: eFigure 1 Supplement 2 Flow chart of enrollment. ECMO extracorporeal membrane oxygenation; VV veno-venous; VA veno-arterial. [file 13054_2020_3270_MOESM7_ESM.docx]

79,668,156 patients from 1700 academic hospitals

Operation of ECMO or operation code: 39.6500 (n=2087)

Excluded

Unclear gender statement (n=11)

Age unknown（n=3）

Patients enrolled (n=2073)

Identify the type of ECMO support

VV ECMO (n=714)

VA ECMO (n=1359)

**eFigure 1 Flow chart of enrollment**

ECMO extracorporeal membrane oxygenation; VV veno-venous; VA veno-arterial
