# Supplementary material for: Cross-sectional study for the clinical application of extracorporeal membrane oxygenation in Mainland China, 2018
Source: Crit Care. 2020 Sep 11;24:554. doi: 10.1186/s13054-020-03270-1 (PMC7484920; doi:10.1186/s13054-020-03270-1)
Supplement: Supplementary file 8 — Additional file 8: eFigure 2 the number of ECMO cases and in-hospital mortality in different months ECMO extracorporeal membrane oxygenation; VV veno-venous; VA veno-arterial. [file 13054_2020_3270_MOESM8_ESM.docx]

**eFigure 2 The number of ECMO cases and in-hospital mortality in different months**

ECMO extracorporeal membrane oxygenation; VV veno-venous; VA veno-arterial.
